# Mapping and direct valuation: do they give equivalent EQ-5D-5L index scores?

**DOI:** 10.1186/s12955-015-0361-y

**Published:** 2015-10-05

**Authors:** Nan Luo, Yin Bun Cheung, Raymond Ng, Chun Fan Lee

**Affiliations:** Saw Swee Hock School of Public Health, National University of Singapore, Singapore, Singapore; Center for Quantitative Medicine, Duke-NUS Graduate Medical School, Singapore, Singapore; Department of Biostatistics, Singapore Clinical Research Institute, Singapore, Singapore; Department of International Health, University of Tampere, Tampere, Finland; Department of Medical Oncology, National Cancer Center, Singapore, Singapore

## Abstract

**Objective:**

Utility values of health states defined by health-related quality of life instruments can be derived from either direct valuation (‘valuation-derived’) or mapping (‘mapping-derived’). This study aimed to compare the utility-based EQ-5D-5L index scores derived from the two approaches as a means to validating the mapping function developed by van Hout et al for the EQ-5D-5L instrument.

**Methods:**

This was an observational study of 269 breast cancer patients whose EQ-5D-5L index scores were derived from both methods. For comparing discriminatory ability and responsiveness to change, multivariable regression models were used to estimate the effect sizes of various health indicators on the index scores. Agreement and test-retest reliability were examined using intraclass correlation coefficient (ICC). Whenever appropriate, the 90 % confidence intervals (90 % CI) were compared to predefined equivalence margins.

**Results:**

The mean difference in and ICC between the valuation- and mapping-derived EQ-5D-5L index scores were 0.015 (90 % CI = 0.006 to 0.024) and 0.915, respectively. Discriminatory ability and responsiveness of the two indices were equivalent in 13 of 15 regression analyses. However, the mapping-derived index score was lower than the valuation-derived index score in patients experiencing extreme health problems, and the test-retest reliability of the former was lower than the latter, for example, their ICCs differed by 0.121 (90 % CI = 0.051 to 0.198) in patients who reported no change in performance status in the follow-up survey.

**Conclusion:**

This study provided the first evidence supporting the validity of the mapping function for converting EQ-5D-5L profile data into a utility-based index score.

## Introduction

Utility values of health outcomes described by health-related quality of life (HRQoL) instruments are convenient quality-of-life weights for calculation of quality-adjusted life years (QALYs) in cost-utility analysis (CUA) of health services and technologies [1]. Such values are either directly measured using a valuation technique such as time trade-off or standard gamble (i.e., the valuation approach) or estimated using a mapping function from known utility values of health outcomes defined by another HRQoL instrument (i.e., the mapping approach). For example, the valuation approach has been taken to determine the utility values of the health outcomes or states defined by the EQ-5D-3L questionnaire [2], and the availability of the EQ-5D-3L utility values in turn has stimulated the application of the mapping approach to many HRQoL instruments whose utility values are unknown [3]. Typically, a mapping function is developed by modeling cross-sectional data collected using both the source and target instruments from a group of subjects. The ordinary least-square model is most frequently used in mapping studies [4].

The EQ-5D-5L is a new HRQoL instrument [5] for which van Hout et al. [6] have developed a mapping function based on its relationship with the EQ-5D-3L. Developed from the EQ-5D-3L, the EQ-5D-5L describes similar health states as the EQ-5D-3L. Both instruments describe a respondent's health in terms of mobility, self-care, usual activities, pain/discomfort, and anxiety/depression. While the EQ-5D-3L describes problems in each dimension into 3 levels (no problems, some/moderate problems, and unable/extreme problems), the EQ-5D-5L describes into 5 levels (no problems, slight problems, moderate problems, severe problems, and unable/extreme problems). Therefore, the EQ-5D-3L is an ideal source instrument for the EQ-5D-5L to map into [4]. For estimating the mapping function, 3691 individuals with conditions of varying severity recruited from six European countries were surveyed using both the EQ-5D-5L and EQ-5D-3L questionnaires. The mapping function is based on a nonparametric model for its simplicity and superior fit to the data [6]. For any respondent of the EQ-5D-5L, this mapping function gives the corresponding probabilities of the EQ-5D-3L response patterns (each pattern corresponds to a health state). The utility value of the respondent’s health, which is the corresponding EQ-5D-5L health state, can be calculated as the sum of the known utility values of the EQ-5D-3L health states weighted by the corresponding probabilities. As the EQ-5D-5L utility values determined through the valuation approach were not available at the time when van Hout et al. conducted their mapping study, the resultant mapping function has been recommended as the interim approach to deriving utility values from EQ-5D-5L data [7].

Mapping is regarded as a ‘second-best’ approach to deriving utility values, although it is accepted by NICE as a legitimate approach to generating utility values for CUA [8]. Studies found that mapping functions predict values less extreme than the values which they are used to map into [9, 10] and when they are validated in external datasets [9, 10], suggesting the issue of prediction bias. van Hout et al’s mapping function may be less susceptible to this issue because it was not based on a linear regression model. Fayers and Hays pointed out that any regression model would predict less extreme values because of the phenomenon of regression to the mean [11]. Nevertheless, the performance of this mapping function has not been investigated, to the best of our knowledge.

The objective of this study was to validate van Hout et al’s mapping function for the EQ-5D-5L. The validation was through comparing the measurement properties of the utility values generated using van Hout et al’s mapping function (hereafter referred to as ‘mapping-derived’ index) and those determined through direct valuation of the EQ-5D-5L health states using a time trade-off technique [12] (hereafter referred to as ‘valuation-derived’ index) in patients with breast cancer. We hypothesized that values derived from the two approaches would have equivalent measurement properties and result in comparable QALYs in CUA and thus van Hout et al’s mapping function is a valid approach to generating EQ-5D-5L utility values.

## Materials and methods

### Design and recruitment

This study was approved by the Singapore Health Services Institutional Review Board. Patients were recruited from two sites, namely, the specialist outpatient clinics of the National Cancer Centre, Singapore, and the oncology wards of the Singapore General Hospital. Eligibility criteria were: histologically confirmed breast cancer; 21 years old or above; ability to understand Chinese or English or both; no evidence of brain metastasis, psychosis or severe depression; and willingness to give informed consent. Patients answered an identical Chinese or English questionnaire package according to their preference. Each package included the EQ-5D-5L self-report questionnaire, the Functional Assessment of Cancer Therapy - Breast (FACT-B) questionnaire [13, 14], and questions assessing demographic and performance status. Performance status was assessed using a Likert rating scale ranging from 0 (without symptoms) to 4 (bedridden), excluding the score 5 (death) which is not applicable in this study [15]. This measure is strongly associated with cancer patients’ quality of life, and can be self-administered [16, 17]. The questionnaire package was self-administered or by a research assistant upon request. Interviewer administration is often unavoidable in practice and is allowed by developers of both instruments (http://www.euroqol.org, accessed Jan 19, 2015; http://www.facit.org, assessed Jan 19, 2015). Patients’ managing oncologists also assessed the patients using the above-mentioned performance scale. Other clinical information was retrieved from medical records.

All patients were sent a similar questionnaire package for completion one to two weeks after the baseline survey. In addition to the health-status and performance scales, the follow-up survey package included a question assessing the change in health status using a 7-point Likert scale ranging from ‘very much better’ to ‘very much worse’. Up to two reminders with the questionnaire package were sent to non-respondents.

### Instruments

The EQ-5D-5L contains five questions, each assessing one health dimension including mobility, self-care, usual activities, pain/discomfort, and anxiety/depression. Each question asked respondents to describe their health status on the day of survey as one of five levels including ‘no problems’, ‘slight problems’, ‘moderate problems’, ‘severe problems’, and ‘unable to (do)’ (for mobility, self-care, and usual activities) or ‘extreme problems’ (for pain/discomfort and anxiety/depression) [5]. The questionnaire also includes a vertical, hash-marked visual analogue scale (EQ-VAS) anchored by 0 (the worst imaginable health state) at the bottom and 100 (the best imaginable health state) on the top for respondents to rate their overall health. The English and Chinese versions of the EQ-5D-5L questionnaire for use in Singapore are very similar in wording to their counterparts in the UK and China, and exhibited similar measurement properties [18, 19]. The EQ-5D-5L has been found to have better psychometric properties than the EQ-5D-3L in many patient populations [20–24].

The FACT-B is a breast cancer-specific health-related quality of life instrument of the Functional Assessment of Chronic Illness Therapy Measurement System for chronic diseases. The English and Chinese FACT-B version 4 consist of 37 items that are divided into five subscales (physical, social/family, emotional, functional well-beings, and additional concerns for breast cancer) [13, 14]. Each item is rated on a 5-point Likert scale. Negatively worded items were recoded so that a higher score indicates a better health-related quality of life. The FACT-B total score is the sum of scores of all five subscales, ranging from 0 to 144, while the FACT-General (FACT-G) total score is the sum of scores of the physical, social/family, emotional, functional well-beings subscales, ranging from 0 to 108. Missing values are imputed by the half-rule [25]. The validity and reliability of, and the comparability between the English and Chinese versions of the FACT-B have been previously demonstrated [19, 26].

### Statistical analysis

The valuation-derived EQ-5D-5L index score was calculated using an algorithm recently developed by Ramos-Goñi et al. [12]. This algorithm was developed using utility values of 86 EQ-5D-5L health states directly measured from a general population sample (*N* = 1000) in Spain using both the time trade-off and the discrete choice experiment methods. The two valuation methods were described in detail elsewhere [27]. Briefly, in time trade-off, a series of questions each asking a respondent to choose between a shorter but healthier life and a longer life in an impaired health state; in discrete choice experiment, respondents were asked to answer a set of independent questions each requiring the respondents to indicate preference between two multi-dimensional health states whose superiority is not obvious (e.g., one health state featured by pain and the other featured by depression). The EQ-5D-5L index scores generated using this algorithm range from −0.224 to 1, with 0, 1, and negative values corresponding to death, full health, and health states worse than death, respectively.

The mapping-derived EQ-5D-5L index score was calculated using van Hout et al’s mapping function [6] and the Spanish EQ-5D-3L utility values [28]. The EQ-5D-3L values were determined through the direct valuation approach from a general population sample (*N* = 1000) in Spain using a time trade-off method very similar to the one used in the abovementioned EQ-5D-5L valuation study. The Spanish EQ-5D-3L values range from −0.654 to 1, with 0, 1, and negative scores corresponding to death, full health, and health states worse than death, respectively. An Excel-based calculator developed by the EuroQol Research Foundation (Bas Janssen, personal communication) was used to perform the calculation.

The means scores and their standard deviations of the two indices were compared for the entire sample and subgroups of patients with different demographic and clinical characteristics. The agreement of the two indices was examined using the intraclass correlation coefficient (ICC) and Bland-Altman plot, and the equivalence of the two indices was assessed by comparing the 90 % confidence interval (CI) of the differences with the pre-defined equivalence margin of 0.05. The 90 % CI [29] and the equivalence margin of ±0.05 [30, 31] were chosen to be consistent with previous studies.

The discriminatory power, responsiveness to change and test-retest reliability of the two indices were compared. For discriminatory power, the two EQ-5D-5L indices were simultaneously regressed on a health indicator in a bivariate regression model. Health indicators included oncologist-assessed and self-assessed performance status, FACT-B and FACT-G total scores, EQ-VAS, current evidence of disease and chemo- or radiotherapy. The effect size was quantified by the regression coefficients, β_Mapping_ and β_Valuation_. The estimate of the coefficients together with the correlation between them were used to construct a 90 % CI for the difference in effect size, β_Valuation_-β_Mapping_.

Similarly, for responsiveness to change, the effect size was estimated by regressing the change in the EQ-5D-5L index scores using both value sets from baseline to follow-up on the change in a health indicator in a bivariate regression model. The 90 % CI for the difference in effect size was estimated and compared with the equivalence margin. Only patients who reported in the follow-up survey a change in performance or health status were included in the responsiveness analysis. To deal with the potential regression-to-the-mean effect, analyses were adjusted for the baseline scores. This is a recommended approach for analysis of change in clinical trials [32]. Moreover, the change in scores between baseline and follow-up surveys was compared between the two indices to assess equivalence. As change in utility scores in clinical trials is used to calculate quality-adjusted life years in cost-utility analysis, equivalence in changed scores rather than absolute scores of the two indices in baseline or follow-up determines whether the two indices would lead to equivalent results when used in economic evaluations.

For test-retest reliability, ICC of the two EQ-5D-5L indices was calculated using patients who returned the follow-up questionnaire within 30 days after the baseline survey and reported no change in performance or health status at follow-up. Since the two indices are highly correlated, a 90 % CI for the difference in ICC was constructed using the method proposed by Ramasundarahettige et al. [33]. In the comparison of reliability, we also adopted an equivalence margin of ±0.05. Bland-Altman plots [34, 35] were also generated to assess the test-retest reliability of the two indices.

## Results

**Fig. 1:**
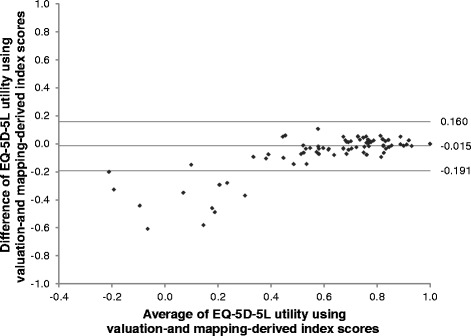
Bland-Altman plots of baseline EQ-5D-5L utility using valuation- and mapping-derived index scores

A total of 280 patients completed the baseline survey. Two patients with missing values and nine proxy-administered patients were excluded, leaving 269 patients. Table 1 summarizes their demographic and clinical characteristics. The mean (standard deviation) score of the valuation- and mapping-derived EQ-5D-5L indices for the sample was 0.811 (0.186) and 0.796 (0.250), respectively, at baseline (Table 2). The difference of 0.015 (90 % CI = 0.006 to 0.024) was small in magnitude and the 90 % CI totally fell within the equivalence margin of ±0.05, indicating equivalence of the two indices. Both indices attained the maximum value of 1, while the lowest score was −0.111 and −0.370 for the valuation- and mapping-derived index, respectively. The ICC between the two indices was 0.915. Their Bland-Altman plot shows that, for the 12 patients (4.5 %) with the lowest health utility value in the sample, the mapping-derived index score (mean = −0.118, range = −0.370 to 0.118) was apparently smaller than the valuation-derived index score (mean = 0.261, range = −0.111 to 0.487) (Fig. 1). All the 12 patients experienced extreme problems in at least one EQ-5D-5L dimension.

**Table 1:** Patient characteristics at baseline (*N* = 269)

Characteristics	N	(%)
Age, mean (standard deviation)	52.1	(9.9)
Language version		
English	169	(62.8)
Chinese	100	(37.2)
Race		
Chinese	221	(82.2)
Malay	25	(9.3)
Indian	19	(7.1)
Others	4	(1.5)
Marital status		
Married	185	(69.3)
Single	54	(20.2)
Divorced/separated	15	(5.6)
Widowed	13	(4.9)
Education level		
Primary or below	69	(25.7)
Secondary	120	(44.8)
Postsecondary	79	(29.5)
Oncologist-assessed performance status		
0	140	(52.2)
1	93	(34.7)
2	24	(9.0)
3 or above	11	(4.1)
Patients’ self-assessed performance status		
0	102	(37.9)
1	128	(47.6)
2	20	(7.4)
3 or above	19	(7.1)
Patient type		
Inpatient	89	(33.1)
Outpatient	180	(66.9)
Current evidence of disease (present)	142	(53.2)
Purpose of visit		
Treatment - adjuvant/curative/hormone therapy	118	(44.4)
Treatment - palliative	98	(36.8)
Follow up (no treatment)	50	(18.8)
On chemotherapy/radiotherapy (yes)	116	(43.1)
Mode of interview		
Self-administration	244	(90.7)
Interview-administration	25	(9.3)

**Table 2:** Baseline EQ-5D-5L index scores by demographic and clinical characteristics

Characteristic	Mean (standard deviation)		Difference (90 % confidence interval)
	Valuation-derived index score	Mapping-derived index score	
All patients	0.811 (0.186)	0.796 (0.250)	0.015 (0.006 to 0.024)^a^
Language version			
English	0.815 (0.182)	0.808 (0.226)	0.007 (−0.001 to 0.015)^a^
Chinese	0.804 (0.192)	0.774 (0.285)	0.029 (0.009 to 0.050)^a^
Race			
Chinese	0.816 (0.189)	0.799 (0.258)	0.017 (0.007 to 0.028)^a^
Malay/Indian/others	0.788 (0.169)	0.782 (0.206)	0.006 (−0.010 to 0.022)^a^
Marital status			
Married	0.826 (0.174)	0.816 (0.291)	0.010 (0.000 to 0.020)^a^
Single/divorced/widowed	0.774 (0.207)	0.745 (0.304)	0.028 (0.009 to 0.048)^a^
Education level			
Primary or below	0.793 (0.219)	0.762 (0.304)	0.031 (0.008 to 0.054)
Secondary	0.818 (0.178)	0.799 (0.249)	0.019 (0.004 to 0.033)^a^
Post-secondary	0.817 (0.168)	0.820 (0.192)	−0.003 (−0.011 to 0.005)^a^
Oncologist-assessed performance status			
0	0.889 (0.120)	0.891 (0.140)	−0.002 (−0.008 to 0.005)^a^
1	0.774 (0.160)	0.766 (0.207)	0.008 (−0.005 to 0.022)^a^
2	0.612 (0.224)	0.514 (0.360)	0.098 (0.038 to 0.158)
3 or above	0.575 (0.349)	0.457 (0.507)	0.118 (0.025 to 0.210)
Patients’ self-assessed performance status			
0	0.917 (0.104)	0.917 (0.127)	−0.000 (−0.008 to 0.007)^a^
1	0.796 (0.132)	0.796 (0.160)	0.000 (−0.009 to 0.009)^a^
2	0.739 (0.159)	0.728 (0.182)	0.011 (−0.008 to 0.030)^a^
3 or above	0.421 (0.271)	0.214 (0.419)	0.207 (0.128 to 0.285)^b^
Patient type			
Inpatient	0.704 (0.228)	0.650 (0.338)	0.054 (0.029 to 0.078)
Outpatient	0.864 (0.133)	0.868 (0.146)	−0.004 (−0.008 to 0.001)^a^
Current evidence of disease			
Absent	0.875 (0.132)	0.878 (0.151)	−0.003 (−0.010 to 0.004)^a^
Present	0.753 (0.208)	0.721 (0.294)	0.032 (0.016 to 0.047)^a^
On chemotherapy/radiotherapy			
Yes	0.755 (0.181)	0.730 (0.272)	0.025 (0.007 to 0.043)^a^
No	0.854 (0.179)	0.845 (0.219)	0.008 (0.000 to 0.016)^a^
Mode of administration			
Self-administered	0.820 (0.175)	0.807 (0.236)	0.013 (0.004 to 0.022)^a^
Interviewer-administered	0.722 (0.257)	0.684 (0.342)	0.038 (0.003 to 0.073)

^a^Equivalence was confirmed

^b^Non-equivalence was confirmed

The valuation-derived index score was generally higher than the mapping-derived index score in patients with similar characteristics (Table 2). However, the magnitude of the difference was small and the corresponding 90 % CI fell within the pre-specified equivalence margin for most of the patient groups. The only non-equivalence was observed in patients with a performance status score of 3 or above (*N* = 19, difference = 0.207, 90 % CI = 0.128 to 0.285), the majority of whom (*N* = 14) reported severe or extreme problems in at least one of the EQ-5D-5L dimensions.

Table 3 compares the effect size of the two indices for detecting a difference in the performance status, evidence of disease, treatment status, FACT-B and FACT-G total score, and EQ-VAS. For the latter three indicators, the effect size was presented as the change in EQ-5D-5L utility index per 20-point increment in the indicator which is sufficiently large to represent a change in health status. For each indicator, the mapping-derived index score showed a larger effect size than the valuation-derived index score. However, those statistically significant differences were small. For 5 out of 7 indicators, the 90 % CI fell within the pre-specified equivalence margin of ±0.05, thus confirming equivalence.

**Table 3:** Discriminative ability of the two EQ-5D-5L indices

Health indicator	Effect size (standard error)		Difference in effect size (90 % confidence interval)
	Valuation-derived index score	Mapping-derived index score	
Oncologist-assessed performance status	0.115 (0.012)	0.153 (0.016)	−0.038 (−0.048 to −0.027)^a^
Patient’s self-assessed performance status	0.141 (0.010)	0.188 (0.014)	−0.047 (−0.057 to −0.038)
Current evidence of disease (present vs absent)	0.122 (0.022)	0.156 (0.029)	−0.035 (−0.053 to −0.017)
On chemotherapy/radiotherapy (yes vs no)	0.099 (0.022)	0.115 (0.030)	−0.016 (−0.035 to 0.002)^a^
FACT-B total score (per 20-point increment)	0.112 (0.008)	0.135 (0.012)	−0.023 (−0.031 to −0.015)^a^
FACT-G total score (per 20-point increment)	0.134 (0.010)	0.165 (0.015)	−0.031 (−0.041 to −0.021)^a^
EQ-VAS (per 20-point increment)	0.100 (0.010)	0.124 (0.014)	−0.023 (−0.032 to −0.014)^a^

^a^Equivalence was confirmed

In the follow-up survey, 263 (93.9 %) patients returned completed questionnaires. After excluding patients with missing responses to the EQ-5D-5L questionnaire (*N* = 1) or the self-assessed performance status question (*N* = 13), 78 and 129 patients who reported a change in performance and health status, respectively, were included in the analysis of responsiveness to change. Among those, 26 and 108 patients reported improved performance and health status, respectively. The mean changed score for those with improved performance status was 0.044 and 0.060 for the valuation- and mapping-derived indices, respectively (difference = −0.016, 90 % CI = −0.028 to −0.003); for those with improved health status, the corresponding mean changed scores were 0.002 and 0.008 (difference = −0.006, 90 % CI = −0.014 to 0.003). On the other hand, 52 and 21 patients reported worsened performance and health status, respectively. The mean deterioration in patients with worsened performance status was −0.096 and −0.071 based on the valuation- and mapping-derived indices, respectively (difference = 0.015, 90 % CI = −0.055 to 0.025); the mean deterioration for patients with worsening health status was −0.151 and −0.168 based on the two indices (difference = 0.017, 90 % CI = −0.076 to 0.110).

Table 4 presents the effect size for detecting a change in the health indicators, adjusted for the corresponding EQ-5D-5L utility index score at baseline. Similar to that for discriminatory ability, although the mapping-derived score showed a larger effect size than the valuation-derived score for most indicators, the 90 % CI was within the equivalence margin for all the health indicators.

**Table 4:** Responsiveness to change of the two EQ-5D-5L index scores

Health indicator	Effect size (standard error)		Difference in effect size (90 % confidence interval)
	Valuation-derived index score	Mapping-derived index score	
Reported a change in performance status (*N* = 78)			
Change in self-assessed performance status	0.064 (0.016)	0.071 (0.025)	−0.007 (−0.025 to 0.011)^a^
Change in FACT-B total score (per 20-point increment)	0.106 (0.024)	0.109 (0.038)	−0.003 (−0.030 to 0.024)^a^
Change in FACT-G total score (per 20-point increment)	0.119 (0.027)	0.126 (0.042)	−0.007 (−0.037 to 0.024)^a^
Change in EQ-VAS (per 20-point increment)	0.097 (0.022)	0.108 (0.034)	−0.011 (−0.035 to 0.014)^a^
Reported a change in health status (*N* = 129)			
Patient’s self-rated change in health status	0.044 (0.007)	0.055 (0.011)	−0.010 (−0.019 to −0.002)^a^
Change in FACT-B total score (per 20-point increment)	0.081 (0.018)	0.081 (0.029)	0.001 (−0.019 to 0.020)^a^
Change in FACT-G total score (per 20-point increment)	0.101 (0.020)	0.107 (0.029)	−0.007 (−0.029 to 0.015)^a^
Change in EQ-VAS (per 20-point increment)	0.114 (0.017)	0.142 (0.026)	−0.028 (−0.048 to −0.008)^a^

^a^Equivalence was confirmed

**Fig. 2:**
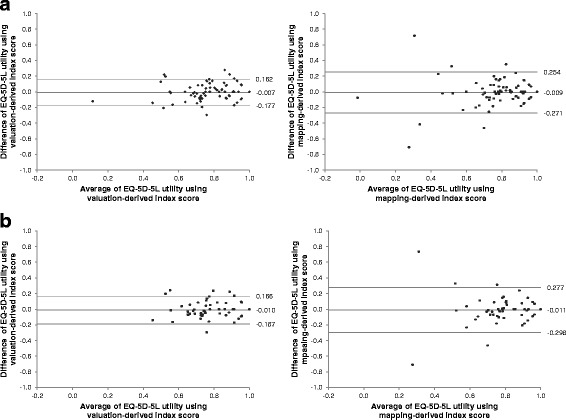
Bland-Altman plots of the baseline and follow-up EQ-5D-5L index derived from direct valuation and mapping in patients who reported no change in **a** self-assessed performance status and **b** health status

In patients who reported no change in performance status (*N* = 138), the ICC was 0.832 and 0.710 for the valuation- and mapping-derived index score, respectively, resulting in a difference of 0.121 (90 % CI = 0.051 to 0.198). In patients reporting no change in health status (*N* = 92), the respective ICCs were 0.793 and 0.607; the difference was 0.186 (90 % CI = 0.078 to 0.307). There was no overlap between the CIs and the equivalence margin of −0.05 to 0.05, indicating non-equivalence. Bland-Altman plots confirm the better test-retest reliability of the valuation-derived index score than the mapping-derived score in both groups of patients (Fig. 2). Take patients who reported no change in performance status for example, the 95 % limits of agreement for the mapping-derived score were wider and 2 patients (1.4 %) in relatively poor health status had dramatically different baseline and follow-up scores based on the mapping approach. One of these two patients improved from ‘unable to walk about’ at baseline to ‘moderate problems in walking about’ at follow-up, with the corresponding change in score being 0.717 for the mapping-derived index and 0.221 for the valuation-derived index; the other patient deteriorated from ‘slight problems in performing usual activities’ at baseline to ‘unable to perform usual activities’ at follow-up, with the corresponding score change being −0.709 and −0.141 for the mapping- and valuation-derived indices, respectively.

## Discussion

In this study, we found that the EQ-5D-5L indices derived from the mapping and direct valuation approaches are largely equivalent in patients with breast cancer. The sensitivity of the two indices to difference and change in health status defined by other measures are mostly equivalent, their score values are equivalent or exchangeable for the study sample and most of its subgroups. Moreover, the two indices exhibited very similar magnitude of change in utility among patients who experienced change in performance or health status. These results provide strong evidence for the validity of van Hout et al’s mapping function for use in both clinical research and economic evaluations. Since the EQ-5D-5L values based on direct valuation may not be available in the near future, our study is an important reference to users of the EQ-5D-5L mapping function.

The good performance of van Hout et al’s mapping function is not surprising since the health dimensions covered by the two EQ-5D questionnaires are identical. The only difference is the (number of) levels used in the two descriptive systems. Moreover, this function does not take the form of a regression model and therefore avoids the prediction bias caused by regression to the mean, an issue observed in mapping functions using a regression model [9, 36–38] and recently criticized by Fayers and Hays [11].

It should be noted, however, the over-time reliability of the mapping-derived EQ-5D-5L index score was poorer than the valuation-derived index score, although the reliability of both indices is considered good based on the psychometric criterion of ICC > 0.70. The lower reliability means that larger measurement errors are contained in the index score, which was also suggested by the greater score dispersion in the baseline survey. Larger measurement error or data variability would lead to greater uncertainty in results of statistical analysis, for example, the larger standard errors of the effect size measure for the mapping-derived index (see Tables 3 and 4). This issue can be solved by increasing the sample size. Nevertheless, we found that the reliability of the mapping-derived index may be poor among individuals who experience extreme health problems. This should be due to the fact that the utility values for such health states are lower according to the EQ-5D-3L value set. As a result, the change in index scores corresponding to health-state transitions between extreme problems and other level of problems based on the EQ-5D-3L value set is larger than that based on the EQ-5D-5L value set. The possibly poor reliability may also suggest an issue of the mapping function itself. It is possible that patients experiencing extreme problems were insufficiently represented in the study sample used to develop the mapping function, and therefore the predictive errors are large for extremely impaired health states.

Another issue associated with the use of the mapping function is lower utility values for patients experiencing extreme health problems, as compared to the value set estimated from direct valuation. The difference could be due to the prediction bias of the mapping function. It is more likely due to the fact that the lowest possible EQ-5D-3L value (−0.654) is much lower than that of EQ-5D-5L (−0.224), although the corresponding health states are identical. The magnitude of the difference suggests that it is unlikely purely due to random measurement errors in the EQ-5D-3L and EQ-5D-5L valuation studies. Rather, it should be due to the time trade-off techniques used in the two Spanish valuation studies. Health states worse than dead were measured using the conventional and lead-time time trade-off techniques [27], respectively, in the Spanish EQ-5D-3L and EQ-5D-5L valuation studies. Although there are no head-to-head comparisons of the utility values measured using the two time trade-off variants, the nuance of the different time trade-off questions and prompts used in the two valuation studies might have somehow caused systematic difference in the resultant utility values. How much this issue impacts on the validity of the mapping function in empirical studies would depend on the proportion of extremely poor health states in the study sample. In the case of the present study where less than 5 % of the sample experienced extreme health problems, the overall equivalence between the two value sets was not affected.

The two issues of the mapping function caution its use in individuals experiencing extreme health problems or study samples comprising a larger proportion of such individuals. In such circumstances, the mapping-derived EQ-5D-5L index score would be neither reliable nor equivalent to the index score derived from an EQ-5D-5L valuation study.

A major limitation of this study is that the EQ-5D-5L data (Singapore), the mapping function (multiple European countries), and the directly measured utility values (Spain) were from different populations. Noise could have been introduced into our analysis because of these differences. Nevertheless, for countries where EQ-5D values are not available, it is a common practice to apply the values from another country. For example, prior to the development of the Singaporean EQ-5D-3L values, the Japanese and UK values were applied in Singapore [22, 23]. Moreover, it is unlikely that Spanish patients with breast cancer would respond to the EQ-5D-5L questionnaire in a very different way than their counterparts in Singapore. Nevertheless, the findings of our study may not be generalizable to other patient populations or existing value sets of other countries. Hence, studies such as the present one should be conducted with new patient samples and other valuation-derived EQ-5D-5L value sets once available to further assess this mapping function.

In summary, the mapping function developed by van Hout et al. is likely to generate equivalent EQ-5D-5L utility values to those derived from direct valuation of the EQ-5D-5L health states in patients with breast cancer. The mapping-derived EQ-5D-5L index score can be as discriminative and responsive as the index score using directly measured utility values. The performance of this mapping function in other patient populations should be assessed in future studies.
